# Novel 1,2,3-Triazole Erlotinib Derivatives as Potent IDO1 Inhibitors: Design, Drug-Target Interactions Prediction, Synthesis, Biological Evaluation, Molecular Docking and ADME Properties Studies

**DOI:** 10.3389/fphar.2022.854965

**Published:** 2022-05-23

**Authors:** Gui-Qing Xu, Xiao-Qing Gong, Ying-Ying Zhu, Xiao-Jun Yao, Li-Zeng Peng, Ge Sun, Jian-Xue Yang, Long-Fei Mao

**Affiliations:** ^1^ Henan Engineering Research Center of Chiral Hydroxyl Pharmaceutical, School of Chemistry and Chemical Engineering, Henan Normal University, Xinxiang, China; ^2^ College of Chemistry and Chemical Engineering, Lanzhou University, Lanzhou, China; ^3^ Key Laboratory of Agro-Products Processing Technology of Shandong Province, Key Laboratory of Novel Food Resources Processing Ministry of Agriculture, Institute of Agro-Food Science and Technology Shandong Academy of Agricultural Sciences, Jinan, China; ^4^ The Third Affiliated Hospital of Guangzhou University of Chinese Medicine, Guangzhou, China; ^5^ Department of Neurology, The First Affiliated Hospital of Henan University of Science and Technology, Luoyang, China; ^6^ School of Nursing, Henan University of Science and Technology, Luoyang, China

**Keywords:** erlotinib, 1,2,3-triazole, DTI, cell assay, docking, ADME analysis

## Abstract

Indoleamine 2,3-dioxygenase 1 (IDO1) plays a predominant role in cancer immunotherapy which catalyzes the initial and rate limiting steps of the kynurenine pathway as a key enzyme. To explore novel IDO1 inhibitors, five derivatives of erlotinib-linked 1,2,3-triazole compounds were designed by using a structure-based drug design strategy. Drug-target interactions (DTI) were predicted by DeePurpose, an easy-to-use deep learning library that contains more than 50 algorithms. The DTI prediction results suggested that the designed molecules have potential inhibitory activities for IDO1. Chemical syntheses and bioassays showed that the compounds exhibited remarkable inhibitory activities against IDO1, among them, compound **e** was the most potent with an IC_50_ value of 0.32 ± 0.07 μM in the Hela cell assay. The docking model and ADME analysis exhibited that the effective interactions of these compounds with heme iron and better drug-likeness ensured the IDO1 inhibitory activities. The studies suggested that compound **e** was a novel and interesting IDO1 inhibitor for further development.

## Graphical Abstract

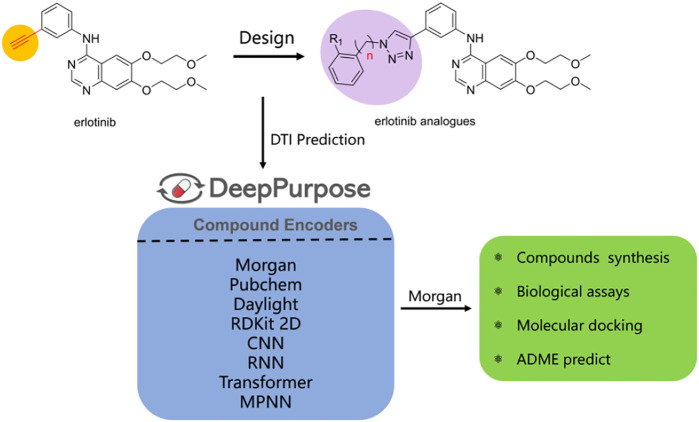

## 1 Introduction

**FIGURE 1:**
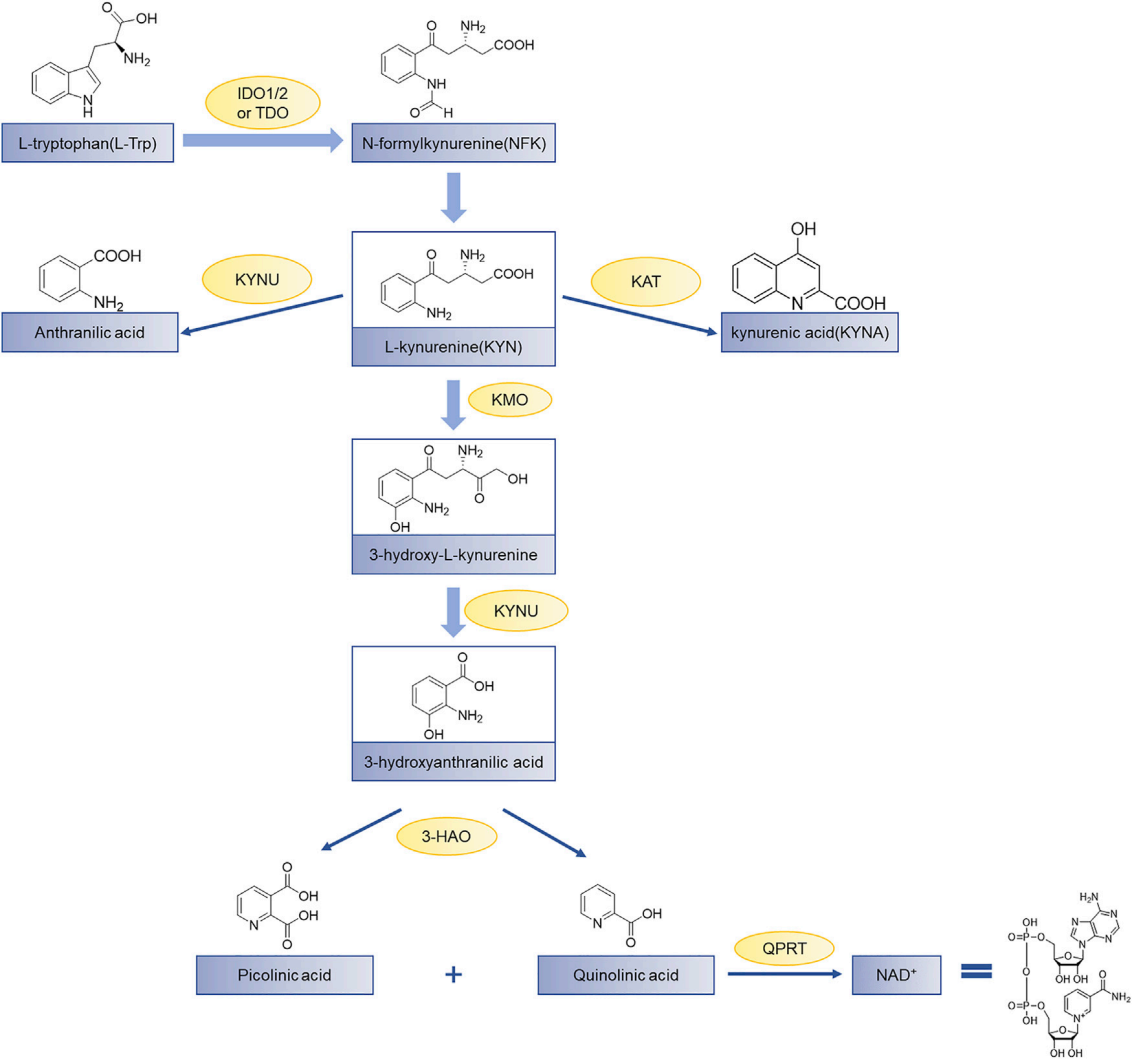
The kynurenine pathway of tryptophan metabolism. Tryptophan (Trp) is used for protein synthesis, while also participates in the metabolism of a series of important signaling molecules. The kynurenine pathway of Trp metabolism is catalyzed by three different enzymes to produce N-formylkynurenine, including indoleamine 2,3-dioxygenase 1 (IDO1), indoleamine-2,3-dioxygenase 2 (IDO2), and tryptophan 2,3-dioxygenase (TDO); KAT, kynurenine aminotransferase; KMO, kynurenine-3-monooxygenase; KYNU, kynureninase; 3-HAO, 3-hydroxyanthranilate 3,4-dioxygenase; QPRT, quinolinic-acid phosphoribosyl transferase (Zhai et al., 2018).

Indoleamine 2,3-dioxygenase 1 (IDO1) is a heme-containing enzyme that catalyzes the initial and rate-determining steps of the kynurenine pathway (Batabyal & Yeh, 2007; Coletti et al., 2017; andDong et al., 2021). Over 95% of tryptophan (Trp) metabolism occurs through the kynurenine pathway and produces a variety of catabolites with various biological activities (Cheong et al., 2018). Tryptophan in humans is mainly degraded to produce N-formylkynurenine (NFK) by a series of catabolic enzymes, indoleamine 2,3-dioxygenase 1 (IDO1), indoleamine-2,3-dioxygenase 2 (IDO2), and tryptophan 2,3-dioxygenase (TDO) (Wang et al., 2019), the product NFK from which it is rapidly metabolized to L-kynurenine. Finally, the final product of the kynurenine pathway is the coenzyme nicotinamide adenine dinucleotide (NAD^+^) which participates in the redox reaction in the metabolic pathway (Figure 1). The up-regulated kynurenine pathway leads to the consumption of Trp and the accumulation of immunosuppressive metabolites, thereby hindering the elimination of cancer cells mediated by immune cells and allows tumors to evade host immune surveillance (Marmarelis and Aggarwal, 2018). Therefore, blocking the kynurenine pathway in a tumor microenvironment is considered a potential strategy for cancer immunotherapy.

**FIGURE 2:**
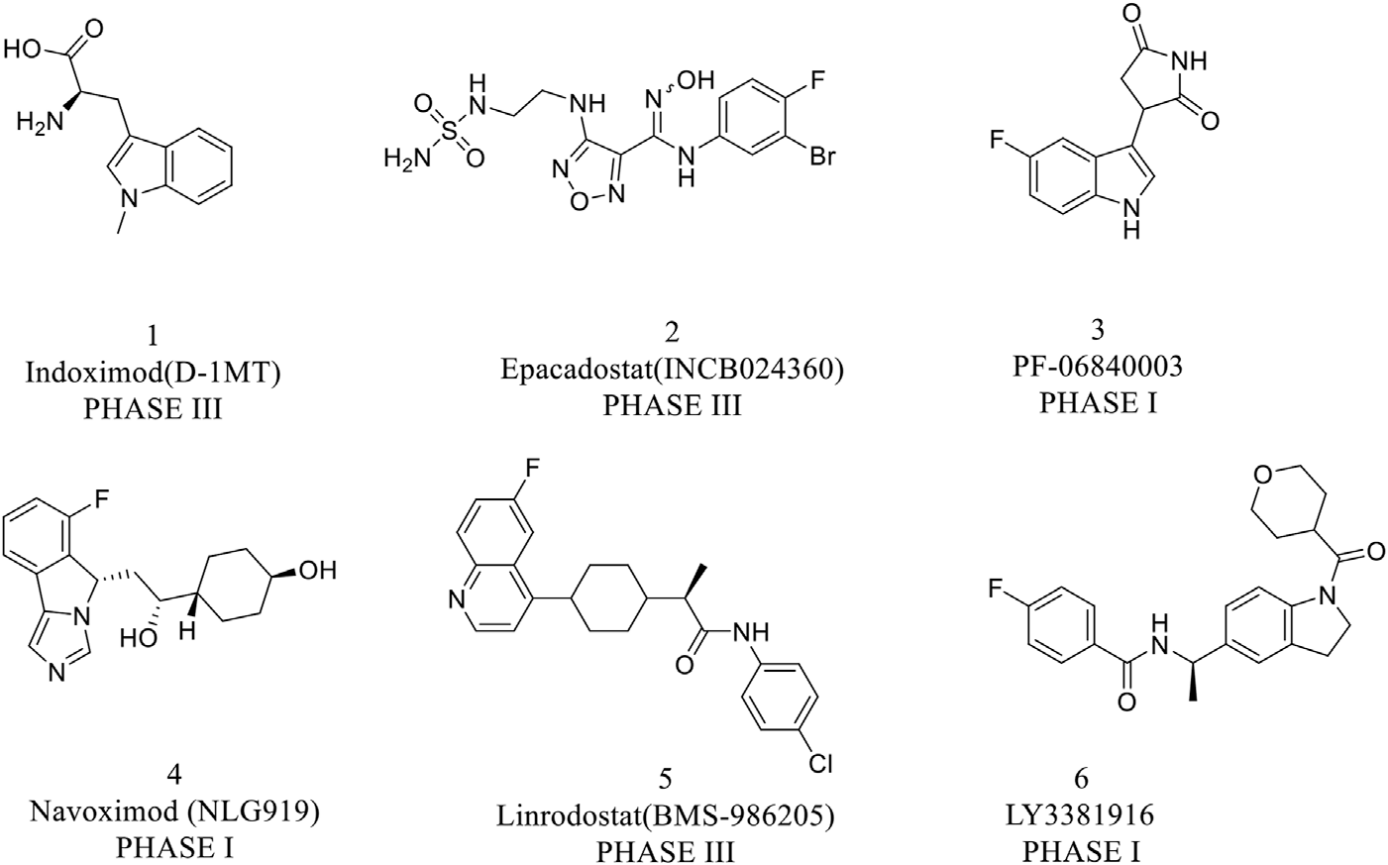
Representative structure of IDO1 inhibitors in clinical trials.

IDO1 is widely expressed in human macrophages and dendritic cells, which has attracted great attention from pharmaceutical academia and industries in the field of tumor immunotherapy (Wu et al., 2021). So far, various IDO1 inhibitors have been reported in the clinical research phase as shown in Figure 2. Indoximod (IC_50_ = 70 nM) is the most representative competitive IDO1 inhibitor developed by NewLink Genetics (Fox et al., 2018). Epacadostat is a highly selective IDO1 inhibitor of N-hydroxyamidine developed by Incyte, which has access to clinical phase III up till the present moment, with an average IC_50_ value of 71.8 and 10 nM in enzymatic and cellular assays, respectively (Yue et al., 2017; Komiya and Huang, 2018). Navoximod (IC_50_ = 28 nM) is a non-competitive IDO1 inhibitor with a 4-phenylimidazole structure developed by NewLink Genetics in the phase I clinical trial (Nayak-Kapoor et al., 2018; Jung et al., 2021). Ma et al. (2019) found that navoximod is quickly absorbed and well tolerated. Although IDO1 inhibitors have made great progress, it is regrettable that the results of clinical trials reported recently are not satisfactory. What’s more, the phase III clinical trial (ECHO-301/Keynote-252) of the fastest-growing IDO1 inhibitor epacadostat combined with PD-1 monoclonal antibody pembrolizumab for the treatment of metastatic melanoma ended in failure. The failure of this clinical trial does not mean that the IDO1 target is not druggable, but it shows that there are indeed many problems to be solved in the development of IDO1 inhibitors (Muller et al., 2019). Therefore, we still need to explore novel structures of IDO inhibitors.

Heterocyclic compounds have a wide range of applications in medicinal chemistry, and about 80% of the drugs on the market contain heterocyclic characteristics. Among them, triazole compounds are the most studied basic components of active molecules, and have shown excellent activity characteristics in anti-tumor, anti-viral, and anti-infective drugs, especially 1,2,3-triazole compounds (Bozorov et al., 2019). The copper catalyzed azide alkyne cycloaddition reaction [Cu-AAC, “click chemistry”] is used for the synthesis of 1,2,3-triazole compounds (Rostovtsev et al., 2002). The compounds introducing 1,2,3-triazole into the terminal alkyne group of erlotinib have an obvious IDO inhibitory activity (Yang et al., 2013). In addition, icotinib-1,2,3-triazole derivatives have a potential IDO1 inhibitory effect (Mao et al., 2020). These facts arouse our interest that the introduction of substituted 1,2,3-triazole into the terminal alkyne group of erlotinib may also improve the IDO1 inhibitory activity.

With the development of artificial intelligence, the affinity prediction of proteins and drugs is widely used in structure-based drug design, including virtual screening, optimization of lead compounds, etc. The affinity prediction model can help us preliminarily predict the activities of the designed compounds, which greatly reduces the time and cost of drug design. DeepPurpose (Huang et al., 2020) is a deep learning library for drug-target interaction predictions. It can achieve more than 50 algorithms by encoding proteins and compounds, including 7 protein encoders and 8 compound encoders. This model performed well on multiple benchmark datasets.

In computer-aided drug design (CADD), molecular docking is widely used for identifying protein–ligand interaction modes. Molecular docking provides useful information about protein–ligand interactions and is frequently used to predict the binding orientation of small molecule drug candidates (Vijesh et al., 2013). The binding modes of the designed compounds and IDO1 were studied by molecular docking. The ability to predict the pharmacokinetic properties of molecules in the early stage of drug discovery can reduce the possibility of failure in the development stage. Therefore, to consider the drug-like properties of the compounds, ADME properties were predicted by employing the QikProp program.

Inspired by the aforementioned studies, it was worthwhile to prepare 1,2,3-triazole compounds based on the erlotinib core structure in a view to get promising anti-cancer agents. Erlotinib is used to treat non-small-cell lung cancer in clinical research. It has a terminal alkyne, which is a good starting point. Molecular docking revealed that the hydrophilic ether chain of erlotinib can enter the hydrophilicity of the IDO1 target. Moreover, we considered that triazole can bind to the heme iron in the IDO1 target and could competitively inhibit the activity of IDO1. Based on these, we designed several triazole compounds based on the erlotinib parent structure. Then, we combined with the DTI prediction model to predict the affinities of molecules for IDO1. Chemical synthesis and biological activity evaluation experiments identified the inhibitory activities of the molecules against IDO1. Molecular docking and ADME analysis further studied the modes of interaction and drug-like properties of the combination of molecules and IDO1. In the current research, we have used this design strategy to obtain IDO1 inhibitors with better activities.

## 2 Materials and Methods

### 2.1 Structure-Based Design Strategy

**FIGURE 3:**
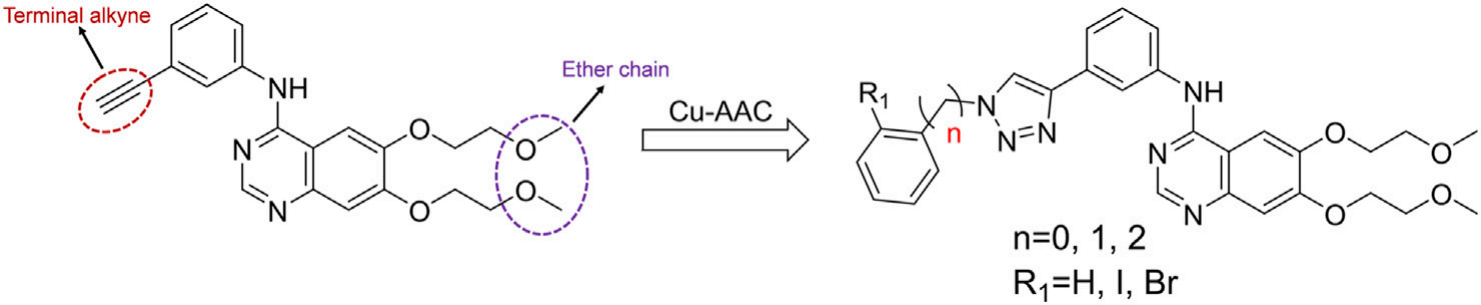
Design of the 1,2,3-triazole series compounds based on erlotinib.

According to the literature, 1,2,3-triazole scaffolds showed prominent enzymatic and cellular IC_50_ values against IDO1, which can effectively inhibit tumor formation. Previous studies have shown that icotinib-1,2,3-triazole derivatives possessed potential IDO1 inhibition (IC_50_ = 0.37 μM). Erlotinib and icotinib are reported to be effective with non-small-cell lung cancer (NSCLC) as Tyrosine kinase inhibitors (TKIs). Moreover, erlotinib exhibited a better treatment efficacy than icotinib in NSCLC (*p* = 0.037) (Xue et al., 2016; Tan et al., 2017). Based on the aforementioned results, we assumed that erlotinib could act on an IDO1 target, and a series of erlotinib-1,2,3-triazole compounds were designed as shown in Figure 3.

### 2.2 Drug–Target Interaction Prediction

**FIGURE 4:**
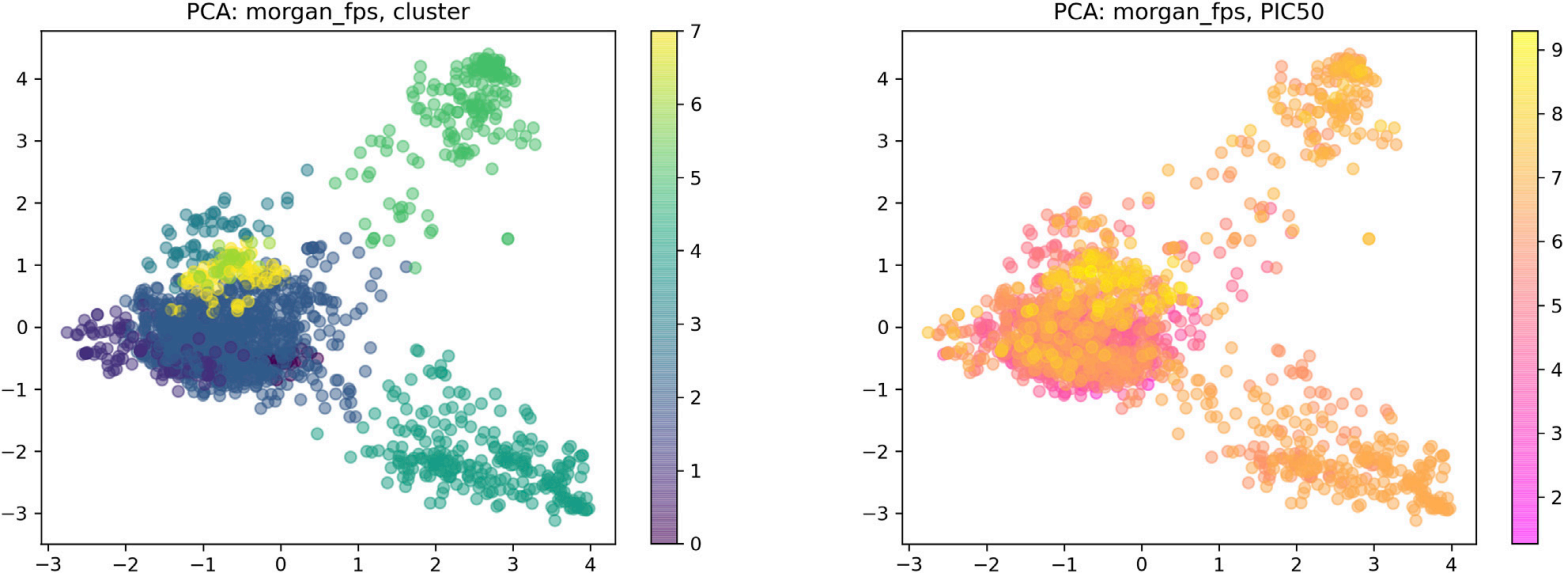
Visual chemical space of the IDO1 database.

Drug–target interaction prediction was implemented by DeepPurpose. First, we constructed an IDO1 dataset containing 1,785 molecules collected from the ChEMBL database. The activity range of these molecules is 0.5–53.9 nM. The IDO1 dataset used IC_50_ as the binding affinity values which were converted to pIC_50_ by Formula 1 (Öztürk et al., 2018). Moreover, we used the principal component analysis (PCA) dimensionality reduction and k-means clustering methods based on the Morgan fingerprints to demonstrate the diversity of the IDO1 database structure as shown in Figure 4. After clustering, the distribution of the data reflected the structural diversity. Among them, there were 92 compounds containing triazole structures. Here, we used a ligand-based method to predict the interactions of molecules and proteins. The SMILES of the compounds as input were then mapped to a vector representation by molecular encoders with a deep transformation function. Compounds were encoded by eight different modalities of encoders, including multi-layer perceptron (MLP) on Morgan, PubChem, Daylight and RDKit 2D fingerprint, convolutional neural network (CNN), recurrent neural network (RNN), transformer, and message passing graph neural network (MPNN). Next, the learned compounds were embedded into an MLP decoder to generate predictions. Finally, we can obtain the binding scores and binary outputs to predict whether the protein binds the compound or not. In this work, we used the Mean Square Error (MSE), Concordance Index, and Pearson Correlation as evaluation indexes to predict DTI.

$$pIC_{50}=-\log_{10}\frac{IC_{50}}{1e^{9}}$$ (1)

### 2.3 Materials and Chemistry

**FIGURE 5:**
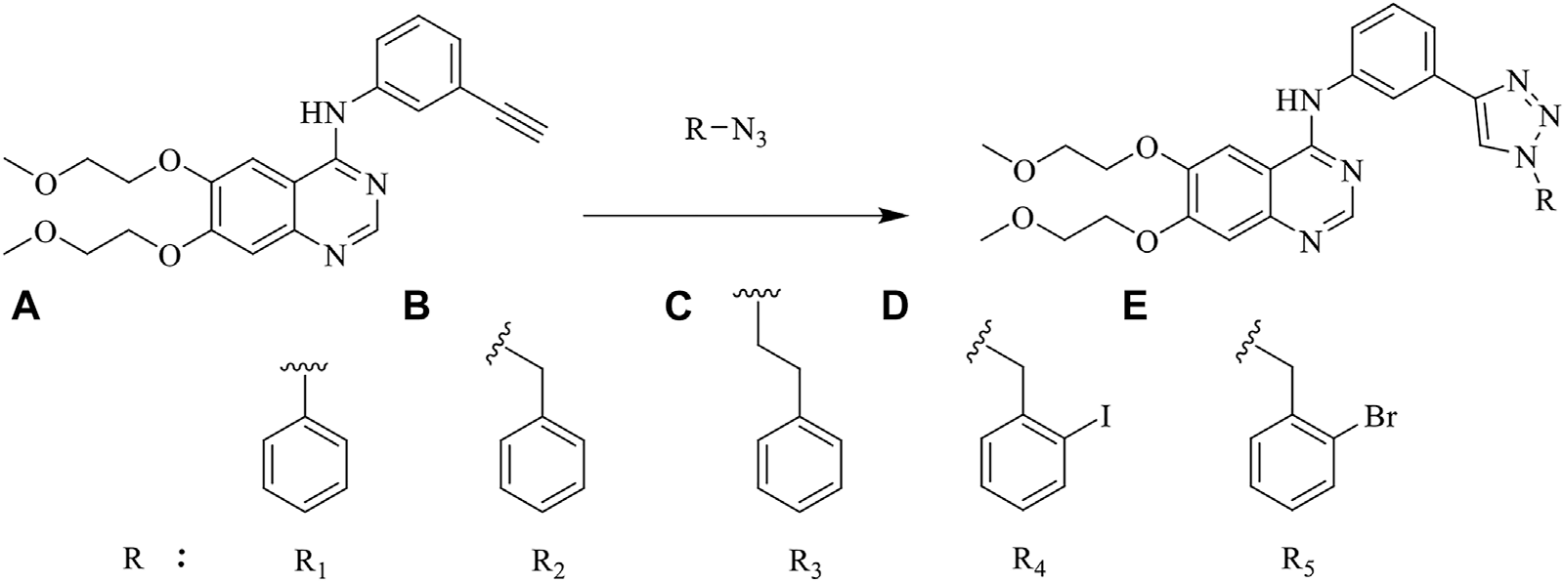
The reaction routes to erlotinib-1,2,3-triazole derivatives.

Erlotinib-1,2,3-triazole derivatives were in-house synthesized. The reaction routes were exhibited in Figure 5. All compounds were purchased from Aladdin’s reagent (China). All reagents and solvents obtained from commercially available sources were used without further purification. ^1^H NMR and ^13^C NMR spectra were acquired in DMSO-d_6_ solution with a Bruker600 spectrometer. Chemical shifts (*δ*) were given in parts per million with tetramethylsilane as the internal reference and coupling constants were expressed in hertz. High-resolution mass spectra (HRMS) measurements were carried out using a Bruker MicrOTOF-Q II mass spectrometer.

Hela cell line, DMEM medium, and fetal bovine serum were purchased from ATCC (VA, United States). Recombinant human IFN-γ was purchased from R&D systems (Emeryville, CA, United States). 3.05 N trichloroacetic acid, 4-(dimethylamino)benzaldehyde, and acetic acid were purchased from Sigma Aldrich (St. Louis, MI, United States).

### 2.4 General Procedure for Preparation of Compound **a**–**e**

Erlotinib (1.0 mmol) and aryl-azido (1.2 mmol) were added to a mixed solvent (water: tert-butanol: THF = 1:1:1, 60 ml). Copper sulfate (0.1 mmol) and sodium ascorbate (0.2 mmol) were added to the mixture and the reaction was stirred at 60°C. After completion of the reaction (monitored by TLC), the mixture was extracted with dichloromethane (20 ml × 5). The combined organic phase was washed successively with water and brine, and then dried with anhydrous sodium sulfate and desolventized. The residue was purified through column chromatography (CH_2_Cl_2_/MeOH = 30:1) to obtain the desired compounds as a crystalline powder.

#### 2.4.1 [6,7-Bis-(2-methoxy-ethoxy)-quinazolin-4-yl]-[3-(1-phenyl-1H-[1,2,3]triazol-4-yl)-phenyl]-amine

m.p. 137–140°C; ^1^H NMR (600 MHz, DMSO-*d*_*6*_): *δ* 9.63 (s, 1H, NH), 9.34 (s, 1H, CH), 8.50 (s, 1H, CH), 8.38 (s, 1H, Ar-H), 7.99 (d, *J* = 7.6 Hz, 2H, Ar-H), 7.94 (d, *J* = 9.8 Hz, 2H, Ar-H), 7.66 (dd, *J* = 16.3, 8.6 Hz, 3H, Ar-H), 7.53 (d, *J* = 18.2 Hz, 2H, Ar-H), 7.24 (s, 1H, Ar-H), 4.34–4.29 (m, 4H, CH_2_CH_2_), 3.81–3.76 (m, 4H, CH_2_CH_2_), 3.39 (s, 3H, CH_3_), 3.36 (s, 3H, CH_3_); ^13^C NMR (150 Hz, DMSO-*d*_*6*_): 156.88, 154.08, 153.42, 148.58, 147.81, 147.45, 140.61, 137.13, 130.99, 130.42, 129.59, 129.21, 122.73, 121.03, 120.49, 120.15, 119.48, 109.44, 108.66, 103.69, 70.61, 70.54, 68.84, 68.51, 58.88, 58.82; HR MS(ESI) m/z: calcd for C_28_H_28_O_4_N_6_Na [M + Na]^+^ 535.2064, found 535.2069.

#### 2.4.2 N-(3-(1-Benzyl-1H-1,2,3-triazol-4-yl)phenyl)-6,7-bis(2-methoxyethoxy)quinazolin-4-amine

m.p. 89–92°C; ^1^H NMR (600 MHz, DMSO-*d*_*6*_): *δ* 9.56 (s, 1H, NH), 8.67 (s, 1H, CH), 8.49 (s, 1H, CH), 8.27 (s, 1H, Ar-H), 7.95–7.86 (m, 2H, Ar-H), 7.56 (d, *J* = 7.7 Hz, 1H, Ar-H), 7.51–7.28 (m, 6H, Ar-H), 7.24 (s, 1H, Ar-H), 5.67 (s, 2H, CH_2_), 4.33–4.29 (m, 4H, CH_2_CH_2_), 3.81–3.75 (m, 4H, CH_2_CH_2_), 3.39 (s, 3H, CH_3_), 3.36 (s, 3H, CH_3_). ^13^C NMR (150 Hz, DMSO-*d*_*6*_): 156.83, 154.06, 153.40, 148.56, 147.44, 147.12, 140.52, 136.50, 131.39, 129.48, 129.30, 128.66, 128.42, 122.28, 122.12, 120.79, 119.23, 109.43, 108.68, 103.69, 70.60, 70.54, 68.83, 68.51, 58.88, 58.82, 53.53; HR MS (ESI) m/z: clad for C_29_H_30_O_4_N_6_Na [M + Na]^+^ 549.2221, found 549.2231.

#### 2.4.3 [6,7-Bis-(2-methoxy-ethoxy)-quinazolin-4-yl]-[3-(1-phenethyl-1H-[1,2,3]triazol-4-yl)-phenyl]-amine

m.p. 109–112°C; ^1^H NMR (600 MHz, DMSO-*d*_*6*_): *δ* 9.56 (s, 1H, NH), 8.53 (s, 1H, CH), 8.49 (s, 1H, CH), 8.24 (s, 1H, Ar-H), 7.93 (s, 1H, Ar-H), 7.89 (d, *J* = 8.9 Hz, 1H , Ar-H), 7.51 (d, *J* = 7.7 Hz, 1H, Ar-H), 7.45 (t, *J* = 7.8 Hz, 1H, Ar-H), 7.29 (t, *J* = 7.4 Hz, 2H, Ar-H), 7.22 (dd, *J* = 13.1, 6.9 Hz, 4H, Ar-H), 4.68 (t, *J* = 7.3 Hz, 2H, CH_2_), 4.33–4.29 (m, 4H, CH_2_CH_2_), 3.81–3.75 (m, 4H, CH_2_CH_2_), 3.38 (s, 3H, CH_3_), 3.36 (s, 3H, CH_3_), 3.24 (t, *J* = 7.3 Hz, 2H, CH_2_); ^13^C NMR (150 Hz, DMSO-*d*_*6*_): 156.84, 154.06, 153.39, 148.56, 147.42, 146.59, 140.51, 138.11, 131.54, 129.48, 129.17, 127.08, 122.19, 121.88, 120.69, 119.17, 109.43, 108.66, 103.68, 70.60, 70.53, 68.83, 68.51, 58.87, 58.82, 51.12, 36.04; HR MS(ESI) m/z: calcd for C_30_H_32_O_4_N_6_Na [M + Na]^+^563.2377, found 563.2381.

#### 2.4.4 [6,7-Bis-(2-methoxy-ethoxy)-quinazolin-4-yl]-{3-[1-(2-iodo-benzyl)-1H-[1,2,3]triazol-4-yl]-phenyl}-amine

m.p. 93–96°C; ^1^H NMR (600 MHz, DMSO-*d*_*6*_): *δ* 9.63 (s, 1H, NH), 8.64 (s, 1H, CH), 8.54 (s, 1H, CH), 8.32 (s, 1H, Ar-H), 8.06–7.90 (m, 3H, Ar-H), 7.63 (d, *J* = 7.7 Hz, 1H, Ar-H), 7.50 (dd, *J* = 16.4, 8.0 Hz, 2H, Ar-H), 7.28 (s, 1H, Ar-H), 7.20 (dd, *J* = 11.8, 7.6 Hz, 2H, Ar-H), 5.75 (s, 2H, CH_2_), 4.37–4.34 (m, 4H, CH_2_CH_2_), 3.85–3.80 (m, 4H, CH_2_CH_2_), 3.43 (s, 3H, CH_3_), 3.41 (s, 3H, CH_3_). ^13^C NMR (150 Hz, DMSO-*d*_*6*_): 156.86, 154.09, 153.34, 148.57, 147.30, 146.93, 140.50, 140.00, 138.36, 131.32, 130.80, 130.17, 129.49, 129.38, 122.52, 122.38, 120.88, 119.30, 109.42, 108.58, 103.72, 99.70, 70.59, 70.54, 68.84, 68.52, 58.88, 58.83, 58.03; HR MS(ESI) m/z: calcd for C_29_H_29_O_4_N_6_INa [M + Na]^+^ 675.1187, found 675.1196.

#### 2.4.5 [6,7-Bis-(2-methoxy-ethoxy)-quinazolin-4-yl]-{3-[1-(2-bromo-benzyl)-1H-[1,2,3] triazol-4-yl]-phenyl}-amine

m.p. 94–97°C; ^1^H NMR (600 MHz, DMSO-*d*_*6*_): *δ* 9.60 (s, 1H, NH), 8.63 (s, 1H, CH), 8.50 (s, 1H, CH), 8.27 (s, 1H, Ar-H), 7.98–7.84 (m, 2H, Ar-H), 7.72 (d, *J* = 7.9 Hz, 1H, Ar-H), 7.58 (d, *J* = 7.7 Hz, 1H, Ar-H), 7.46 (dt, *J* = 11.5, 7.7 Hz, 2H, Ar-H), 7.34 (t, *J* = 8.3 Hz, 1H, Ar-H), 7.29–7.16 (m, 2H, Ar-H), 5.76 (s, 2H, CH_2_), 4.33–4.29 (m, 4H, CH_2_CH_2_), 3.81–3.75 (m, 4H, CH_2_CH_2_), 3.38 (s, 3H, CH_3_), 3.36 (s, 3H, CH_3_). ^13^C NMR (150 Hz, DMSO-*d*_*6*_): 156.88, 154.10, 153.31, 148.58, 147.22, 146.92, 140.47, 135.28, 133.42, 131.32, 131.00, 129.94, 129.50, 128.83, 123.37, 122.53, 122.41, 120.91, 119.33, 109.41, 108.53, 103.72, 87.36, 70.60, 70.53, 68.84, 68.52, 58.88, 58.83, 53.63; HR MS(ESI) m/z: calcd for C_29_H_29_O_4_N_6_BrNa [M + Na]^+^ 627.1331, found 627.1336.

### 2.5 Indoleamine 2,3-Dioxygenase 1 Enzymatic Inhibition Assay

To perform the Hela cell based IDO1 assay, Hela cells were seeded at 50,000 cells per well into a 96-well microplate in 100 μl of DMEM complete growth medium overnight. The next day, 100 μl of the diluted inhibitor was added into each well in the growth medium, then human IFN-γ was added with a final concentration of 100 ng/ml. Cells were incubated for 18 h. On the third day, 40 μL of the medium was removed into a new 96-well plate and 20 μl of 3.05 N trichloroacetic acid (TCA) was added. The plate was incubated at 50°C for 30 min to hydrolyze N-formyl kynurenine produced by IDO to kynurenine. The plate was then centrifuged at 2,500 rpm for 10 min to remove sediments. 100 μl of supernatant per well was transferred to another 96-well plate and mixed with 100 μl of 2% (w/v) 4-(dimethylamino) benzaldehyde in acetic acid. The plate was incubated at room temperature for 10 min, the yellow color derived from kynurenine was recorded by measuring the absorbance at 480 nm using a microplate reader (PerkinElmer, United States).

### 2.6 Cell Antiproliferative Activity Assay

Cell anti-proliferative activity to Hela was evaluated by the CCK8 assay. The Hela cells were seeded at a density of 2,000 cells per well into a 96-well microplate in 100 μl of the growth medium. Cells were incubated at 37°C and 5% CO_2_ overnight. The next day, 100 μl per well of the diluted inhibitor in the growth medium was added with the final concentration from 0.25 to 16.0 μM. The cells were treated with DMSO as control. A series of dilutions were made in 0.1% DMSO in the assay medium so that the final concentration of DMSO was 0.1% in all of treatments. Cells were incubated at 37°C and 5% CO_2_ for 48 h. Then, 10 μl of CCK8 was added to each well. The plates were incubated at 37°C for 2 h, after that, the plates were recorded by measuring the absorbance at 450 nm with the reference wavelength of 630 nm using an EnVision Multilabel Reader (PerkinElmer). All assays were conducted with three parallel samples and three repetitions.

Toxicity to SHEE was evaluated by using the MTT assay. The SHEE cells were seeded in 96-well plates with densities of 2,200–2,500 cells/well in 100 μl. One day after seeding, the concentration of the test compounds being between 0 and 50 μM, 0.1% DMSO were added to the cells as control. Approximately 2,200–2,500 transfected cells in 100 μl were incubated in quintuplicate in 96-well plates. After 48 h, MTT was added and incubated in the plate for 1–4 h in the incubator. The absorbance at 490 nm was measured using a microplate reader (Thermo).

### 2.7 Molecular Docking Studies

Molecular docking was performed in a Schrödinger 2015 version of the Glide module (https://www.schrodinger.com), using the co-crystal structure of Amg-1 with human IDO1 (PDB: 4PK5) (Tojo et al., 2014). 4PK5 is the eutectic structure of IDO1 and a triazole inhibitor. The 3D structure of the protein was prepared using the Protein Preparation Wizard module. The structures of compounds **a**–**e** were pre-processed by LigPrep with OPLS-2005 force field. The grid was generated by the Receptor Grid Generation module. The grid box size was 20 Å × 20 Å × 20 Å (Amg-1 is the coordinate center). Heme iron interacts with ligands to form coordination interactions. To ensure the accuracy of the docking result, we generated metal coordination constraints with heme iron ions as the center, and set other parameters as default values. Compounds specific to each target protein were further analyzed for binding free energy perturbation by the molecular mechanics method using Prime MM-GBSA. The protein–ligand interactions were visualized by using the PyMOL software (version 2.4.0).

### 2.8 Absorption, Distribution, Metabolism, Excretion Properties Prediction

Compounds were further subjected to absorption, distribution, metabolism, and excretion (ADME) predictions using the QikProp module of Schrödinger. It is one of the fastest and most accurate tools for a computational compound’s pharmaceutical associated properties. Synthetic compounds were prepared by using the LigPrep module using OPLS_2005 force field algorithm. Then, optimization through energy minimization and determination of ionization states at the specified pH (7 ± 2.0) were conducted. The default parameters were used for ADME prediction in the normal mode (Ibrahim et al., 2021). The following ADME properties were predicted, including partition coefficient (QPlogP octanol/water), predicted aqueous solubility (QPlogS), predicted brain/blood partition coefficient (QPlogBB), gut–blood brain barrier (QPPCaco), predicted IC_50_ value for blockage of HERGK^+^ channels (QPlogHERG), and predicted value of binding to human serum albumin (QPlogKhsa) (Toppo et al., 2021; Tan et al., 2021).

## 3 Results and Discussion

### 3.1 Chemistry

As shown in Figure 5 the target compounds were prepared by Cu-AAC reaction from erlotinib and aryl-azido in the catalysis of copper sulfate at 60°C in the presence of a mixed solvent (water: tert-butanol: THF = 1:1:1, 60 ml). All the novel desired compounds are fully characterized by means of ^1^H NMR, ^13^C NMR, and HRMS measurements.

### 3.2 Drug-Target Interactions Prediction Study

To further predict the activities of the designed molecules, we adopted a ligand-based method to train an IDO1 dataset with different encoders. The IDO1 dataset is randomly divided by 8:1:1 (training set: validation set: test set). After training and testing, the performance of the IDO1 dataset on different models is shown in Table 1. We need to adjust the encoders and training parameters. According to the evaluation results, the MLP on Morgan and CNN performed well in IDO1 dataset, especially the Morgan-based MLP model (MSE = 0.467, *p* = 0.827, and CI = 0.822). The IDO1 dataset performed bad between in-message passing graph neural network and transformer. Consequently, we used the Morgan-based MLP model to predict the affinities of the designed molecules against IDO1. The predicted affinities of the molecules are shown in Table 2. The results suggested that the six molecules designed were all active. The affinities of compound **d**, erlotinib, and **e** comprehensively showed better results. The affinity of compound **b** was better than compound **a**, and compound **c** was the worst. Overall, the predictive activities of these molecules were generally active for IDO1.

**TABLE 1:** The prediction performance of the IDO1 dataset on several models.

Model	MSE	*p*	CI
Morgan	0.467	0.827	0.822
PubChem	0.514	0.811	0.806
Daylight	0.505	0.820	0.818
RDKit2D	0.504	0.816	0.818
CNN	0.487	0.817	0.808
RNN	0.696	0.731	0.771
Transformer	1.459	0.295	0.584
MPNN	1.040	0.537	0.694

**TABLE 2:** Affinity prediction results of the designed molecules.

Compound	Morgan-MLP
**a**	5.240
**b**	5.277
**c**	4.922
**d**	5.453
**e**	5.363
Erlotinib	5.463

### 3.3 Indoleamine 2,3-Dioxygenase 1 Inhibitory Activity and Cell Antiproliferative Activity Study

A series of 1,2,3-triazole compounds were synthesized and tested the activity of molecules against human IDO1 in the standard enzymatic assay, a CCK8, and an MTT anti-proliferative assay (Yue et al., 2009). Hela cell was selected because of its expression in native human IDO1 induced with IFNγ (Liu et al., 2010; Malachowski et al., 2016). The human immortalized esophageal epithelial cell line (SHEE) was used in the MTT assay to evaluate the cytotoxicity of erlotinib derivatives. BMS-986205 was used as a positive control with an IC_50_ value of 0.62 nM (Nelp et al., 2018). IDO1 inhibitory and anti-tumor activities of compounds **a**–**e** are shown Table 3. As expected, some compounds displayed remarkable IDO1 inhibitory activities, which were consistent with DTI predictions. First, we analyzed the effects of phenyl, benzyl, and phenethyl on the inhibitory activities of IDO1. The results showed that the inhibitory activity of compound **b** was reaching 0.68 ± 0.42 μM which was superior to erlotinib. Meanwhile, the inhibitory activity of compound **b** on Hela and SHEE cells was more than 16 and 50 μM, respectively. This indicates that compound **b** can selectively inhibit IDO1 without damaging Hela cells. Then, we explored the influence of bromine and iodine atom at the 2-position of benzyl group. The results showed that halogen is beneficial and the IDO1 inhibitory activities of compound **d** and **e** were 0.40 ± 0.14 μM and 0.32 ± 0.07 μM, respectively, especially bromine atom (compound **e**) (Qian et al., 2016).

**TABLE 3:** IDO1 inhibitory and anti-tumor activities of compounds **a**–**e**.

Compound	Structure	IC_50_ (μM)		
		Hela	IDO1	SHEE
**a**	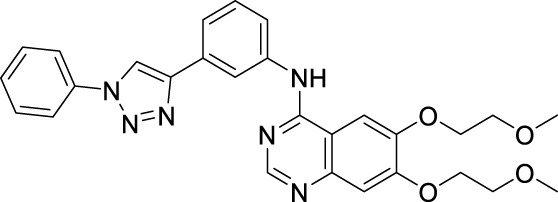	>16	2.61 ± 0.42	14.17 ± 1.61
**b**	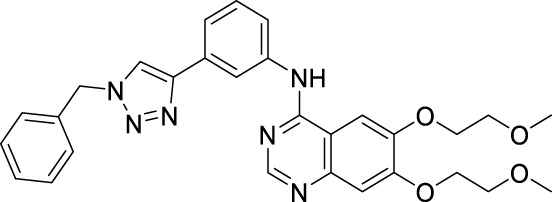	>16	0.68 ± 0.42	>50
**c**	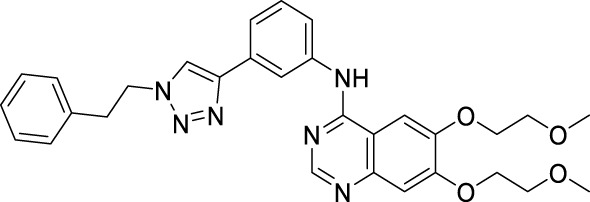	>16	47.14 ± 33.22	33.26 ± 3.43
**d**	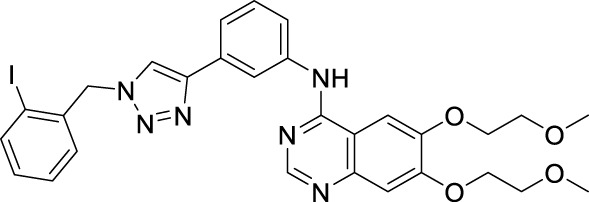	11.50 ± 1.69	0.40 ± 0.14	8.05 ± 0.71
**e**	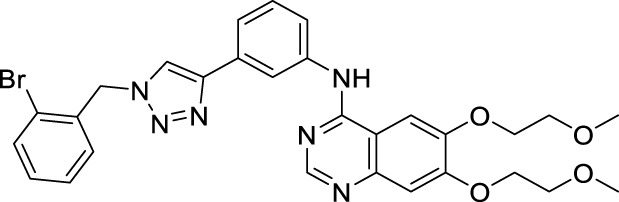	>16	0.32 ± 0.07	22.35 ± 2.53
Erlotinib	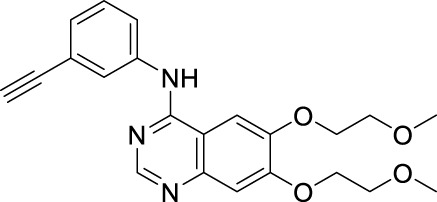	>16	1.95 ± 0.01	20.99 ± 2.11

### 3.4 Molecular Docking Studies

**FIGURE 6:**
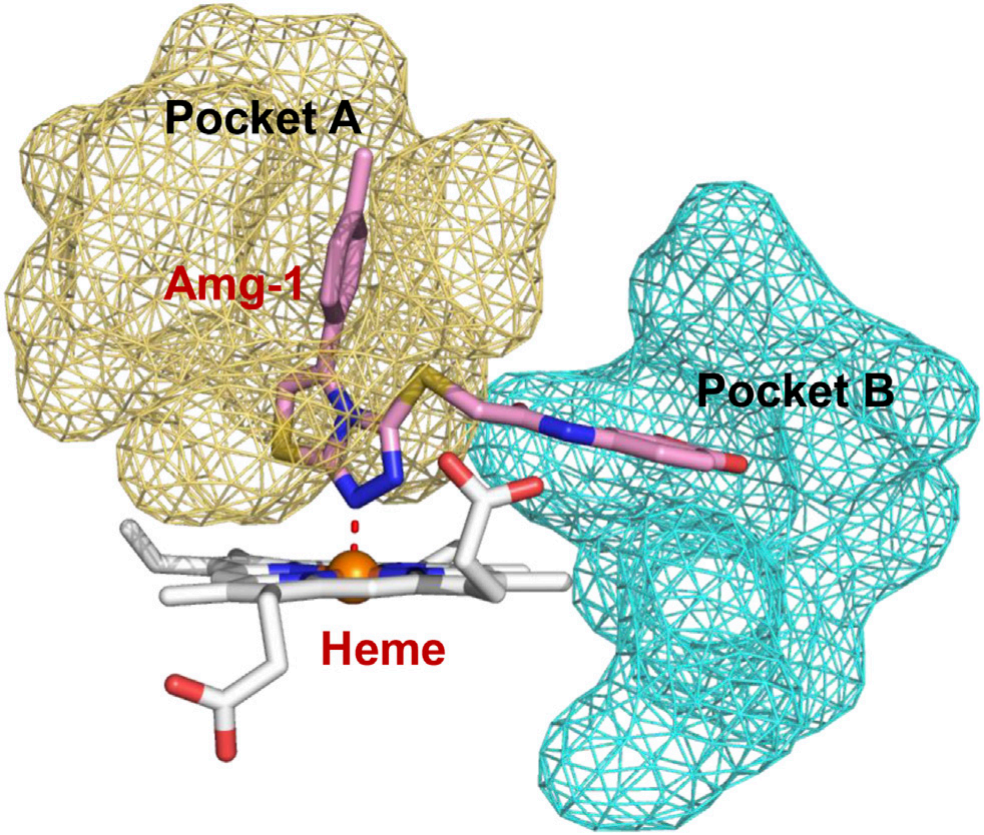
Co-crystallized structure of IDO1/Amg-1 (pink) complex (PDB: 4PK5). Pocket **A** is represented by an yellow surface and pocket **B** is represented by a cyan surface.

**FIGURE 7:**
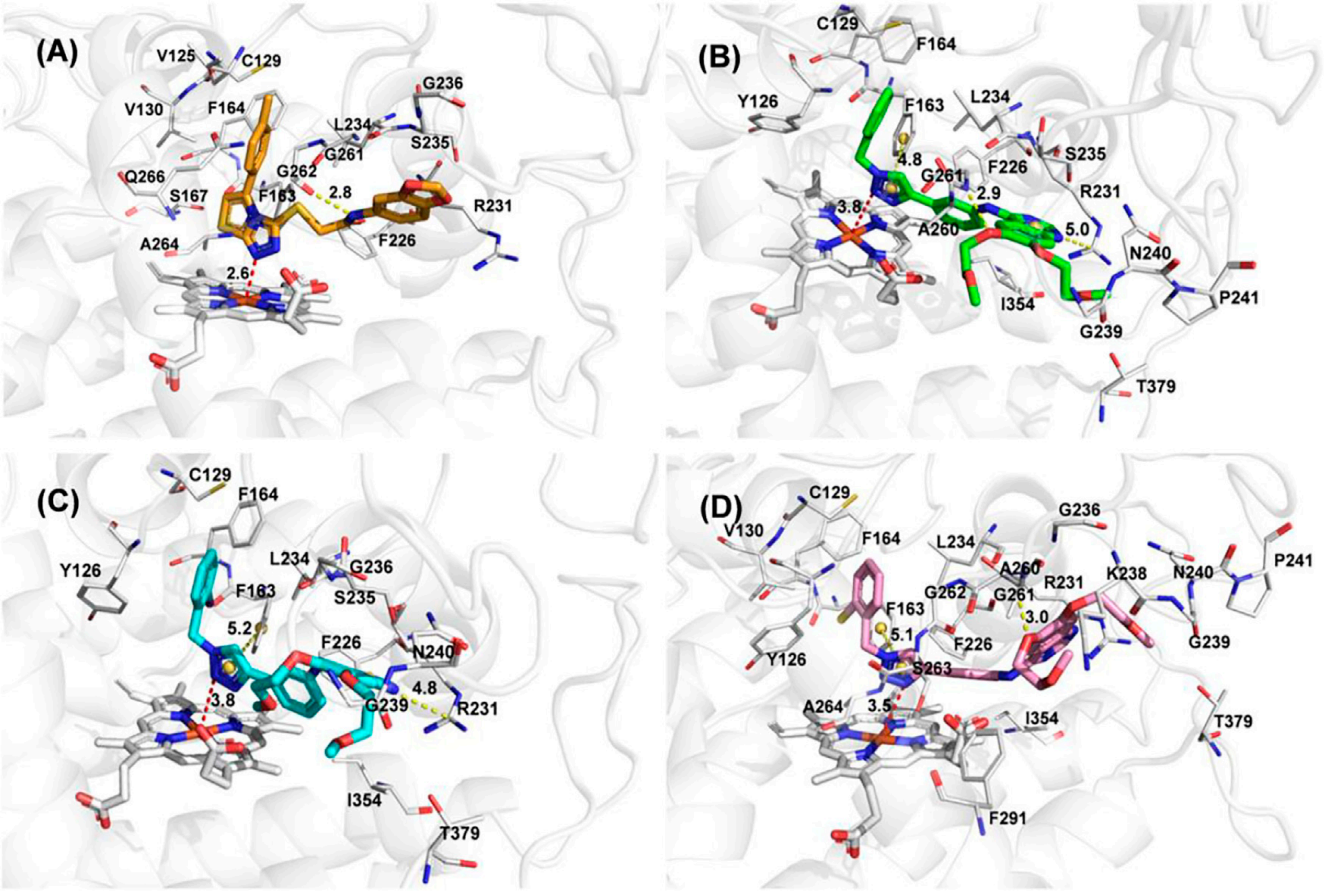
**(A)** The docking conformation of compound Amg-1 (orange) with IDO1. **(B)** The docking binding mode of compound **b** (green) with IDO1. **(C)** The docking binding mode of compound **d** (cyan) with IDO1. **(D)** The docking binding mode of compound **e** (pink) with IDO1.

To better elucidate the potential binding modes and explain the interaction mechanism vividly of the designed compounds with IDO1, we performed docking experiments. The crystal structure of IDO1/Amg-1 complex suggested that the nitrogen of thiazolotriazole in Amg-1 was directly bound to the heme iron to form a metal coordination bond. The p-tolyl group was located on pocket A and the methylenedioxyphenyl was located on the expanded pocket-B (Figure 6). The docking modes of the designed compounds with the enzyme were depicted vividly in Figure 7. From the docking scores of compounds **d** and **e** (Table 4), these two compounds possessed good binding energy. Compounds **d** and **e** could be docked into the hydrophobic site of IDO1 with docking scores of -8.56 and -8.94, respectively.

**TABLE 4:** Molecular docking results of compounds **a–e**.

Compound	Docking score	MMGBSA dGBind (kcal/mol)	Key interactions	Length(Å)
**a**	−6.71	−80.02	H-bonds	2.9
			Lys238:NH_2_-O	
**b**	−8.30	−98.96	H-bonds	2.9
			Gly261:NH_2_-O	
			π − π contact	5.1
			Phe163-triazole	
			π − cation contact	5.0
			Arg231:NH_2_-pyrimidine	
**c**	−5.60	−80.96	None	
**d**	−8.56	−97.23	π − π contact	5.2
			Phe163-triazole	
			π − cation contact	4.8
			Arg231:NH_2_-pyrimidine	
**e**	−8.94	−110.62	H-bonds	3.8
			Gly261:NH_2_-O	
			π − π contact	5.1
			Phe163-triazole	
Erlotinib	−5.64	−72.22	None	

Compound **e** possessed better binding with the heme iron than other designed compounds because of the close distance between the triazole group and the heme iron. Furthermore, the two parts connected to the 1,2,3-triazole ring fit better into the pockets of A and B. The nitrogen of triazole in compound **e** was bound to the heme iron and the o-bromobenzyl group was placed at pocket A. The triazole ring of compound **e** formed π − π interaction with Phe 163, and the 6-methoxyethoxy oxygen atom of quinazoline formed a hydrogen bond interaction with Gly 261. The triazole ring of compound **b** formed π − π interaction with Phe 163, the benzyl group of the triazole ring extended to pocket A, the quinazoline ring occupied pocket B, and the pyrimidine ring of the quinazoline ring formed π −  cation interaction with Arg 231. What’s more, a hydrogen bond interaction was formed with Gly 261 for compound **b**. The binding modes of compounds **d** and **b** were similar except for the hydrogen bond interaction. The docking results of compounds **a** and **c** are shown in Supplementary Figures S1, S2. Compounds **a** and **c** showed poor biological activities. In conclusion, the docking experiments suggested that the interactions of phenyl and phenethyl with protein were weaker than the benzyl group, which was consistent with the activities of the compounds.

### 3.5 Absorption, Distribution, Metabolism, Excretion Studies

ADME analysis was performed to check the drug-likeness and biological properties of the newly discovered IDO1 inhibitors. The ADME properties of the compounds are shown in the Table 5. The aqueous solubility (QPlogS) critical for the estimation of absorption and distribution of the compounds within the body, were ranged between −3.74 and −5.72, respectively. These values were within the acceptable range which is recommended for a molecule to act as a suitable drug. The compounds were predicted to be lipophilic as evidenced by their high QPlogPo/w values. The predicted QPlogKhsa were under the acceptable range of 0.09–0.46. Overall, the predicted ADME properties fit well with the acceptable range, except for the QPloghERG parameter. Blockage of the hERG K^+^ channel can induce cardiotoxicity and result in arrhythmia. The predicted result of compound **e** was in the acceptable range, which further proved that compound **e** was a potential IDO1 inhibitor.

**TABLE 5:** Predicted ADME-related properties of the compounds by Qikprop.

Compound	QPlogPo/W^a^	QPlogS^b^	QPLogBB^c^	QPPCaco^d^	QPlogHERG^e^	QPlogKhsa^f^
**a**	4.35	−3.74	−0.82	1,229.50	−4.82	0.34
**b**	4.91	−5.72	−1.33	883.74	−6.82	0.46
**c**	4.72	−4.19	−1.14	1,069.83	−5.62	0.35
**d**	4.90	−4.91	−0.99	1,122.13	−5.76	0.40
**e**	4.49	−3.75	−0.87	1,149.62	−4.66	0.27
Erlotinib	3.87	−4.09	−0.68	2,681.58	−5.62	0.09

a Predicted water/gas partition coefficient (acceptable range is −2.0–6.5).

b Predicted aqueous solubility, S in mol/L (acceptable range is−6.5–0.5).

c Predicted brain/blood partition coefficient, default is −3.0 to + 1.2.

d Predicted Caco-2 cell permeability in nm/s (<25, poor; >500, great).

e Predicted IC_50_ value for blockage of HERG K^+^ channels (> −5).

f Predicted value of binding to human serum albumin (range is −1.5 to 1.5).

## 4 Conclusion

In this study, a series of erlotinib derivatives containing 1,2,3-triazole rings were designed through a structure-based method, combined with deep learning drug-target interaction models to predict affinities. The derivatives were synthesized and tested for their IDO1 inhibitory activities. The results indicated that the activities of some compounds were better than that of erlotinib. Among them, compound **e**, a structure with an o-bromobenzyl group on the triazole ring exhibited the best IDO1 inhibitory activity (IC_50_ = 0.32 ± 0.07 μM). The docking and ADME characterization revealed that compound **e** was the most potent and drug-like IDO1 inhibitor. In addition, we should also consider the issues of toxicity and oral absorption caused by the triazole ring, and further structural optimization and biological studies of these kinds of compounds are in progress and will be presented in due course.

## Data Availability

The original contributions presented in the study are included in the article/Supplementary Material, further inquiries can be directed to the corresponding authors.
