# Supplementary material for: Novel 1,2,3-Triazole Erlotinib Derivatives as Potent IDO1 Inhibitors: Design, Drug-Target Interactions Prediction, Synthesis, Biological Evaluation, Molecular Docking and ADME Properties Studies
Source: Front Pharmacol. 2022 May 23;13:854965. doi: 10.3389/fphar.2022.854965 (PMC9168369; doi:10.3389/fphar.2022.854965)
Supplement: Supplementary file 1 [file DataSheet1.docx]

**Novel** **1,2,3-****Triazole** **Erlotinib Derivatives as Potent IDO1 Inhibitors: Design, Drug-Target Interactions Prediction, Synthesis, Biological Evaluation, Molecular Docking and ADME Properties Studies**

Gui-Qing Xu^1†^, Xiao-Qing Gong^2†^, Ying-Ying Zhu^1^, Xiao-Jun Yao^2^, Li-Zeng Peng^3^, Ge Sun^4^, Jian-Xue Yang^5,6^*, Long-Fei Mao^1,3^*

^1^Henan Engineering Research Center of Chiral Hydroxyl Pharmaceutical, School of Chemistry and Chemical Engineering, Henan Normal University, Xinxiang, China.

^2^College of Chemistry and Chemical Engineering, Lanzhou University, Lanzhou, China.

^3^Key Laboratory of Agro-Products Processing Technology of Shandong Province, Key Laboratory of Novel Food Resources Processing Ministry of Agriculture, Institute of Agro-Food Science and Technology Shandong Academy of Agricultural Sciences, Jinan, China

^4^The Third Affiliated Hospital of Guangzhou University of Chinese Medicine, Guangzhou, China

^5^Department of Neurology, The First Affiliated Hospital of Henan University of Science and Technology, Luoyang, China.

^6^School of Nursing, Henan University of Science and Technology, Luoyang, China

^†^These authors have contributed equally to this work and share first authorship

*Corresponding authors:

Docyjx1969@126.com

longfeimao1988@163.com

# Figure S1. The docking binding mode of compound a (wheat) with IDO1.


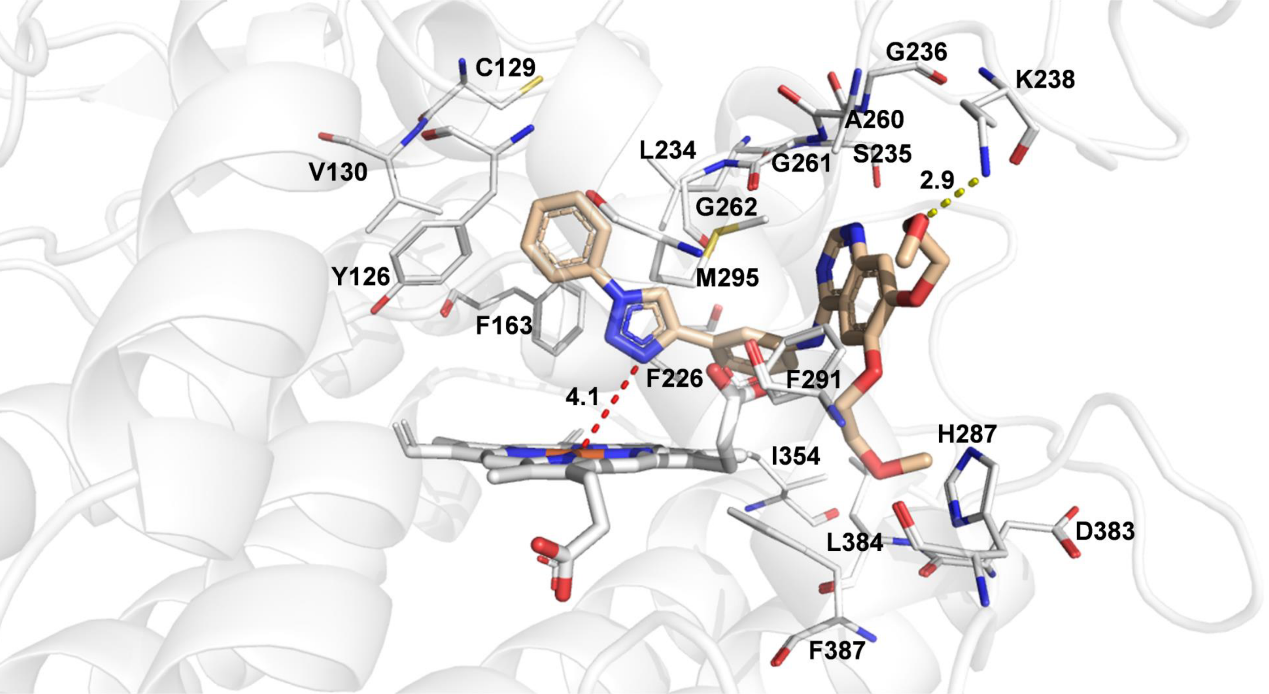


# Figure S2. The docking binding mode of compound c (purple) with IDO1.


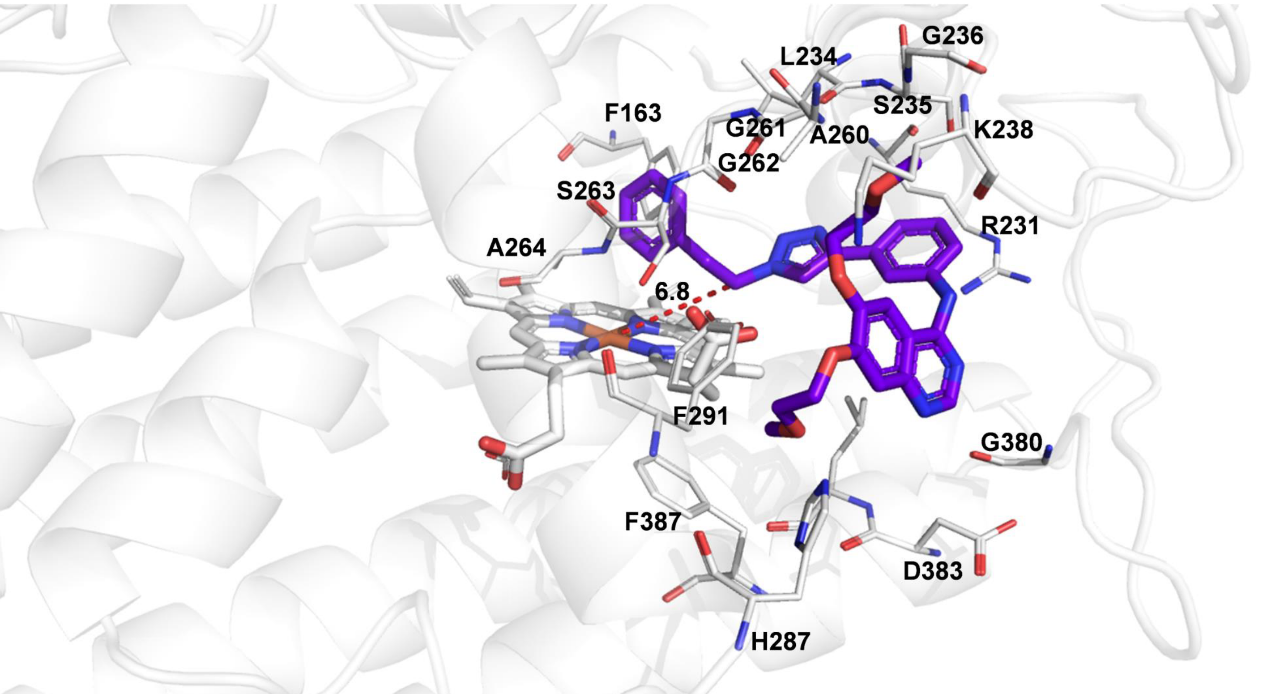


# Figure S3. The docking binding mode of compound erlotinib (pale-yellow) with IDO1.


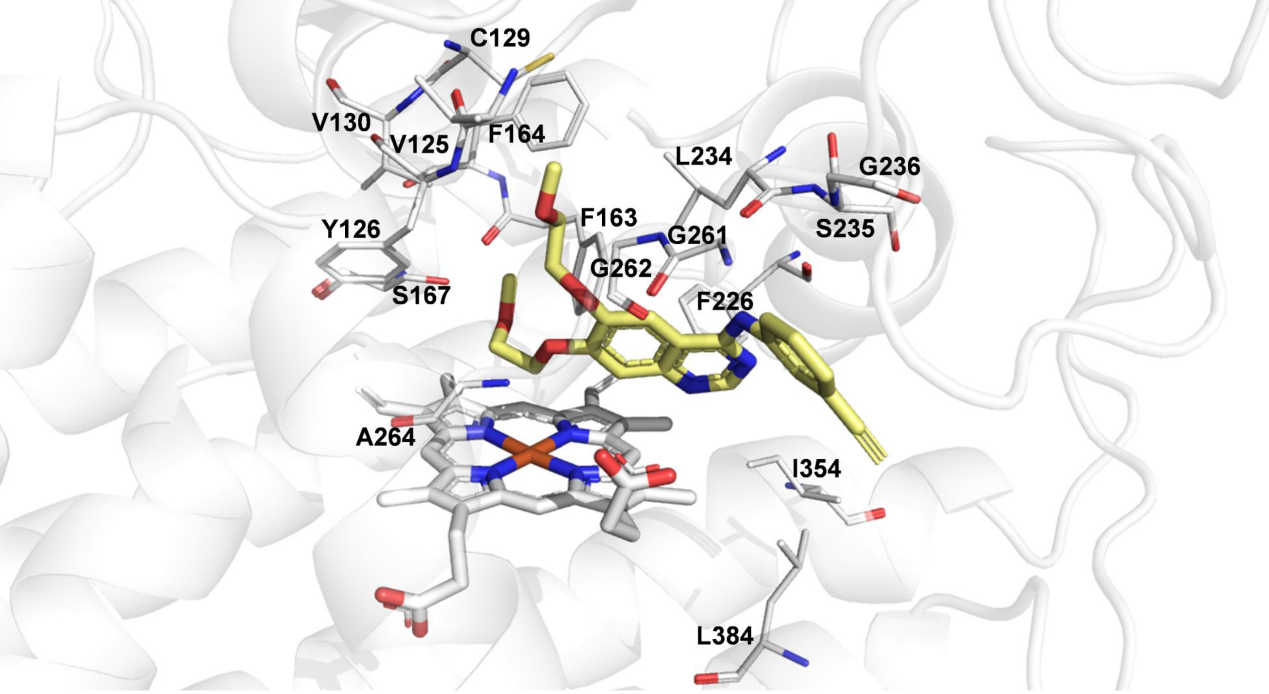


# Figure S4-1. 1H NMR spectrum (600MHz, DMSO-d6) of compound a

# Figure S4-2. ^13^C NMR spectrum (150MHz, DMSO-d_6_) of compound a

# Figure S5-1. ^1^H NMR spectrum (600MHz, DMSO-d_6_) of compound b

# Figure S5-2. ^13^C NMR spectrum (150MHz, DMSO-d_6_) of compound b

# Figure S6-1. ^1^H NMR spectrum (600MHz, DMSO-d_6_) of compound c

# Figure S6-2. ^13^C NMR spectrum (150MHz, DMSO-d_6_) of compound c

# Figure S7-1. ^1^H NMR spectrum (600MHz, DMSO-d_6_) of compound d

# Figure S7-2. ^13^C NMR spectrum (150MHz, DMSO-d_6_) of compound d

# Figure S8-1. ^1^H NMR spectrum (600MHz, DMSO-d_6_) of compound e

# Figure S8-2. ^13^C NMR spectrum (150MHz, DMSO-d_6_) of compound e
